# Burnout status of healthcare workers in the world during the peak period of the COVID-19 pandemic

**DOI:** 10.3389/fpsyg.2022.952783

**Published:** 2022-09-21

**Authors:** Maria Ulfa, Momoyo Azuma, Andrea Steiner

**Affiliations:** ^1^Postgraduate Program, Master of Hospital Administration, Universitas Muhammadiyah Yogyakarta, Yogyakarta, Indonesia; ^2^Department of Infection Control and Prevention, Tokushima University Hospital, Tokushima, Japan; ^3^Department for Occupational Health, Jena University Hospital/Friedrich-Schiller-University, Jena, Germany

**Keywords:** burnout, healthcare workers, COVID-19, psychology, worldwide

## Abstract

During the COVID-19 pandemic, healthcare workers have a high workload and have been exposed to various psychosocial stressors. This study aimed to evaluate health workers during the COVID-19 pandemic in the world. The method used in this research is qualitative with a literature review approach. The data sources in this study were taken from the Scopus database using the keywords “health workers,” “burnout,” AND “coronavirus” from the time range of 2020 to April 25, 2022. From the determination of the time range, 150 documents emerged. This study revealed that the Pearson correlation between total burnout scale scores on healthcare workers, professionals, exhaustion, mental, stress, personal, depression, symptoms, emergency, system, job, and impact indicated that overall burnout scores were associated with depression and anxiety. Stress symptoms had correlation values ranging from 0.84 to 0.73. Job burnout had a significant relationship with exhaustion at 0.89; depression r = 0.73), impacting a score of 0.66. At the same time, the fulfillment of professional and interpersonal disengagement showed a Pearson correlation between the total burnout scale scores on health workers, professionals, exhaustion, mental, stress, personal, depression, symptoms, emergency, system, job, and impact. Overall, the participants in health care workers worldwide experienced high levels of psychological distress. We also found that health workers dealing with COVID-19 pandemic patients were more likely to experience depression, stress, and burnout than health staff who were not personally involved in medical work. Furthermore, this study will be a follow-up study using the Work Ability Index (WAI) to measure work ability and work satisfaction.

## Introduction

Since the end of December 2019, the world has been hit by the COVID-19 pandemic (Sahin et al., 2020; Dobson et al., 2021; Zakaria et al., 2021). It was initially identified in the Chinese city of Wuhan, Hubei Province, in December 2019 and continues to spread today (Luceño et al., 2020; Zakaria et al., 2021). The disease has spread to more than 60 countries worldwide, with more than one million infected patients (Talaee et al., 2022). In addition, coronavirus is the seventh virus proven to infect humans worldwide (El Haj et al., 2020).

Specifically, amid health workers' prolonged struggles against COVID-19 are fatigue (Mollica et al., 2021), chronic psychological conditions with loss of enthusiasm and personal achievement (Pappa et al., 2021), feelings of physical and mental exhaustion (Stone et al., 2021), and depersonalization (Serrão et al., 2021). In this case, the frontline of health professionals has become a significant concern worldwide. Burnout represents work-related tension due to repeated exposure to stressors at work characterized by fatigue (i.e., depletion of one's emotional and physical resources), cynicism (i.e., hostile detachment from work), and reduced efficacy (Cotel et al., 2021; Nishimura et al., 2021). Several studies have been conducted to look at the prevalence and factors associated with burnout. According to Ibar et al. (2021), excessive workload, lack of personal protective equipment, broad media attention, lack of particular care, and negative feelings of support can all add to the mental illness burden of stress (Raudenská et al., 2020). Many factors cause burnout, such as anxiety (Orrù et al., 2021), psychological (Lai et al., 2020; Zhang et al., 2020; Conti et al., 2021; Ferry et al., 2021; Miguel-Puga et al., 2021), high depressive symptoms (Barello et al., 2020), related to uncertainty (Ibar et al., 2021; Zakaria et al., 2021), Stigmatization (Naldi et al., 2021), reluctance to work (Shanthanna et al., 2020; Tiete et al., 2021), and resignation (Ibar et al., 2021; Miguel-Puga et al., 2021; Naldi et al., 2021).

The Agency for Healthcare Research and Quality (AHRQ) defines burnout as “a long-term stress reaction characterized by emotional exhaustion, depersonalization, and a lack of sense of personal accomplishment” (Naldi et al., 2021). Recently, WHO incorporated burnout into ICD-11 (World Health Organization, 2019). In the context of pre-existing high fatigue, the COVID-19 pandemic hit (Mollica et al., 2021). Medical personnel exhaustion has real-world ramifications for medical employees and patients, with significant implications for how the health system responds to current and future outbreaks (Denning et al., 2021). Previous studies have found a series of burnout-related work characteristics in health workers during the SARS and MERS outbreaks. After the SARS outbreak in 2003, hospital healthcare workers who treated SARS patients reported being more tired than hospital healthcare workers who did not handle SARS patients (Maunder et al., 2006). Burnout of health workers was also due to being affected or caused by a lack of resources for treatment during MERS outbreaks (Kim and Choi, 2016).

The result is by Job Demands-Resources Theory (JD-R) (Bakker et al., 2014), showing that Job demands increase tiredness and job resources minimize fatigue. Furthermore, the theory implies that having personal resources reduces burnout. The data from the medical sector support this last theoretical assumption. Under typical working situations, personal resources such as optimism and self-efficacy are connected to decreased fatigue levels in nurses. Based on the JD-R theory and earlier research during the SARS and MERS outbreaks, job demands were assumed to be positively related to burnout. Moreover, during the COVID-19 pandemic, job and personal resources were negatively associated with burnout among healthcare workers. These phenomena provide a 'snapshot' of fatigue, anxiety, depression, and distress among the world's health workers during the COVID-19 pandemic. This action will highlight the need for intervention and identify health professionals more vulnerable to adverse psychological effects. It will help to guide future government and health council decisions about resource allocation for the wellbeing of health workers and better reporting on how these resources might be targeted to those most at risk of adverse mental health outcomes (Ferry et al., 2021).

Based on the background explanation, this study aims to analyze the burnout status of health workers during the peak of the COVID-19 pandemic. It also describes the professional fatigue and physical symptoms of the world's frontline healthcare workers who are directly involved in caring for COVID-19 patients.

## Literature review

### Burnout status of healthcare workers

In explaining the research topic related to the burnout status of health workers, this study used the theoretical basis of Maslach and Jackson (1981) in their theory of the Measurement of Experienced Burnout, explaining burnout syndrome as a syndrome of emotional exhaustion and cynicism that often occurs in working people. Burnout syndrome is also a process in which there is a change in negative behavior in response to pressure and work stress for a prolonged period. In the burnout status of health workers, health workers who experience burnout syndrome will experience loss of enthusiasm or despair, pessimism, make mistakes at work, apathy, get angry easily with patients, and do not want to accept change and lose creativity (Freudenberger, 1974). In addition, burnout syndrome is a general capacity and is described as a multidimensional construct consisting of emotional fatigue, cynicism or depersonalization, and decreased achievement because medical personnel feels excessive emotions, have negative feelings toward their work and have a less sharp sense about work (Jerry and Brodsky, 1981). Social stigmatization, a lack of personal protective equipment, and a high workload on staff can all exacerbate the condition (Jalili et al., 2021). As a result, this pandemic will likely have a significant psychological impact on healthcare workers (Guo et al., 2021).

Furthermore, burnout syndrome occurs due to interpersonal stressors related to work. Maslach and Jackson (1981) provided a fundamental difference between burnout syndrome and stress. According to them, health workers who experience burnout will feel demotivated and hopeless, while health workers who experience stress tend to act emotionally excessively. However, prolonged stress can trigger burnout syndrome, while burnout syndrome conditions experienced by health workers are not necessarily caused by stress. Moreover, burnout syndrome is a process of negative behavior change that occurs in response to stress and work pressure for a long time. Burnout syndrome has also become a psychological phenomenon that counters improving one's performance, effectiveness, and organizational output.

Stressful work requires individual and organizational efforts to cope with burnout syndrome. Burnout syndrome has many factors, such as too heavy a workload. The workload is a burden experienced by medical personnel due to their work. The influence of workload is quite dominant on the performance of human resources but can also harm the safety and health of the workforce. Besides, the workload of health workers is an activity carried out with the type of work and the severity of the work specified in a specific time unit of a health service unit. The workload of health workers includes both physical and mental workload. Too heavy workloads or weak physical abilities can cause workers to suffer from disorders or diseases such as burnout syndrome.

Burnout syndrome can be identified in 11 symptoms, including fatigue and losing energy accompanied by exhaustion. In this case, running from reality is a tool to deny the suffering experienced, boredom, and cynicism, such as feeling no longer interested in the activities they do and even feeling bored and pessimistic about the field of work. It is emotional because, so far, the individual who can do work experiences quickly decreases the ability to do the job soon. The individual who also feels confident in his abilities then experiences feelings of unappreciated, disorientated, psychosomatic problems, suspicion for no apparent reason, depression, and denial of the reality of his situation. Moreover, burnout syndrome has very negative consequences, and burnout can affect a person's physical or mental health, causing psychosomatic disorders, such as mucosal changes, cardiorespiratory conditions, headaches, and others. Psychopathological disorders include anxiety, obsessive-compulsive behavior, depression, and addiction. In addition, the impacts felt by burnout syndrome sufferers encompass physical, psychological, and behavioral.

The explanation related to burnout syndrome concludes that it is a symptom that begins with a process of negative behavior change that occurs in response to stress and work pressure. In this regard, the work environment can determine the occurrence of burnout, such as excessive work, role conflict, the number of individuals who are thirsty to be served, the responsibilities that must be carried out, routine work carried out continuously, role ambiguity, inadequate social support from coworkers, social support from inadequate superiors, low control overwork, and lack of stimulation at work. Moreover, Maslach and Jackson (1981) divided three dimensions of the burnout system: emotional exhaustion (emotional fatigue), depersonalization, and personal accomplishment (self-achievement). Each of these dimensions can be seen in the following figure.

#### Emotional exhaustion

The first dimension of burnout syndrome is emotional exhaustion, where medical personnel feels that they do not want to provide psychological services fully. When medical personnel feels emotional exhaustion, they feel tired even though they have had enough rest and are less enthusiastic about doing activities. Medical personnel who experience burnout syndrome will also avoid or buy time when faced with work that must contact patients. In addition, emotional exhaustion is characterized by prolonged physical, mental, and emotional fatigue. When health workers feel tired, they also tend to behave overextended emotionally and physically, unable to solve their problems, still feel tired even though they have had enough rest and lack the energy to carry out activities (Maslach et al., 2008).

#### Depersonalization

The second dimension of burnout syndrome is depersonalization, characterized by a cynical attitude and a tendency to withdraw from the work environment. This dimension is called depersonalization, which is separating oneself from others, showing cold emotions, and showing adverse reactions to the behavior of others, for example, treating patients less well and getting angry quickly. When medical personnel tends to be cold and keep their distance, they tend not to want to be involved with their work environment. Depersonalization is also a way to avoid disappointment. Negative behavior like this can have a severe impact on work effectiveness.

#### Personal accomplishment

The third dimension is the personal accomplishment of health workers, who have decreased performance, show negative feelings, are not happy, and are not satisfied with their work. Poor self-evaluation results also indicate decreased self-achievement, low interpersonal relationships, loss of enthusiasm, reduced productivity, and lack of adaptability. The decrease in self-achievement is also characterized by feelings of helplessness and the feeling that all the tasks are heavy. When medical personnel feels ineffective, they tend to develop a sense of inadequacy. In addition, every job feels difficult and cannot be done, and self-confidence decreases. The workers become distrustful, and the others distrust them.

## Materials and methods

This research used qualitative methods with a literature study approach that includes several tasks such as gathering library information, reading and recording, and maintaining research materials (Crusswel, 2017). A literature review is also an important activity in research, particularly in academic research, with the primary goal of developing theoretical and practical benefits. Each researcher conducts literature reviews to establish a foundation for gathering and developing a theoretical foundation, a thinking framework, and determining tentative assumptions, often known as research hypotheses. As a result, the researchers may organize, classify, and use a wide range of literature on their subjects.

### Research strategy and selection criteria

This research approach was developed through consensus among all Scopus databases. The authors autonomously distinguished articles published from 2020 until 2022 that reviewed health worker burnout in various countries during the peak of the COVID-19 pandemic through a systematic search of the Scopus database. Each retrieved article with English language constraints was subjected to snowball sampling via reference list search and citation tracking. The following search terms were then used: (“health workers” AND “burnout” AND “coronavirus”). The study population included medical and non-medical health workers in COVID-19-affected nations or regions. From the data searches and data screening results, 150 documents were analyzed.

### Data extraction and quality assessment

The following data were retrieved from each article with two reviews: subject area and keywords. Secondly, data were limited based on the year and English language, making it easier to read and analyze the data. The methods used, limitations, and the total number of participants and their percentage were screened. The authenticity of the retrieved or deliberate data was checked by contrasting the two investigators' collecting forms. The following quality assessment criteria are the representativeness, the size of the sample examined, and the relevancy of the articles concerned.

### Analysis of data

Data analysis in this study utilized the NVivo 12 Plus software and the Vosviewer Software. The NVivo software was used to test the correlation between indicators, variables, and keywords used by previous studies. One finding was then drawn to serve as further research from this correlation. Furthermore, VOSviewer software was employed to map the most dominant keywords when studying burnout health workers. It was to make it easier for further research about burnout health workers.

## Results and discussion

### Analysis of publication output

**Figure 1:**
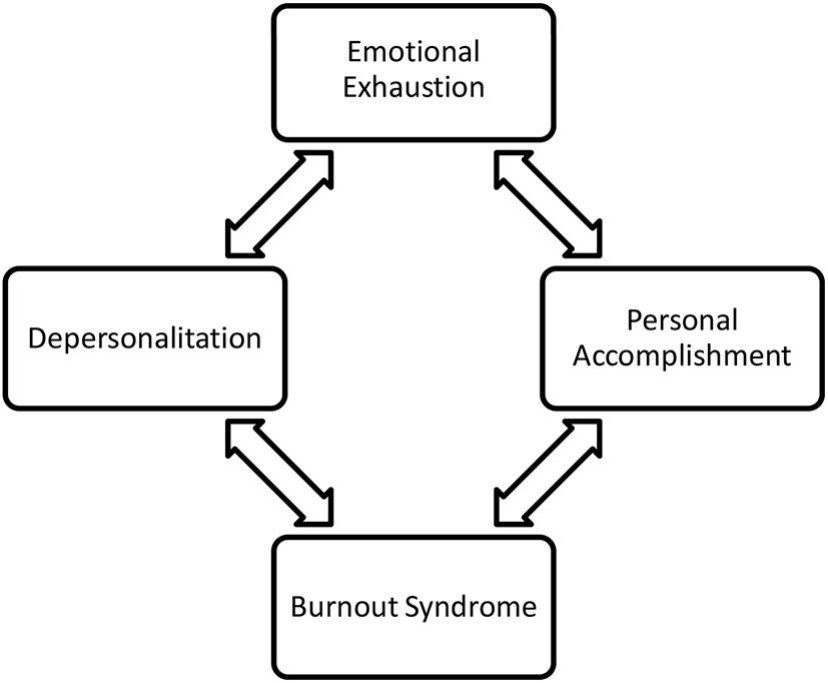
The measurement of experienced burnout. Source: Maslach and Jackson (1981).

In total, 150 publications reviewed the burnout of health workers during the COVID-19 pandemic. The data were taken from the relevant articles and published in 2020 until now. With the acceleration of the COVID-19 pandemic, the highest number of publications was in 2021, as known that 2021 is the peak of the pandemic. This phenomenon invited academics to study and research this phenomenon. To find out the study progress on burnout health workers during the pandemic (Figure 1).

**Figure 2:**
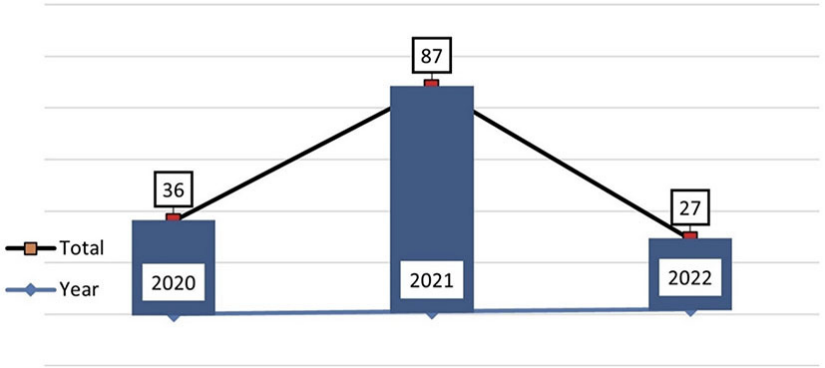
Publication trend of burnout health worker.

The study of burnout health workers in 2021 had the highest number of publications, from January 2020 to May 2021 (Figure 2). They were related that the WHO estimated between 80,000 and 180,000 health workers' deaths worldwide due to COVID-19 (Arbar, 2021). Most of them were doctors and nurses. According to Orrù et al. (2021), the majority of healthcare workers die as a result of the psychological stress, including uncertainty about disease progression (short- and long-term effects), treatment, lack of Personal Protective Equipment (PPE), physical exhaustion, excessive workload, and concerns about direct COVID-19 exposure in the workplace (Britt et al., 2021; Ferreira and Gomes, 2021).

### Scientific production by country

**Figure 3:**
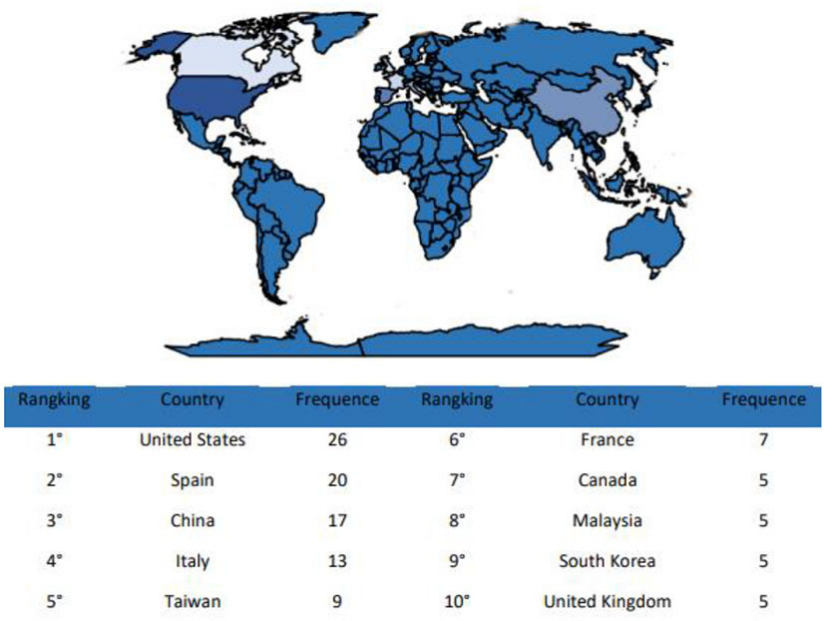
Scientific production by country.

In 48 countries, several publications contributed to the development of studies on the burnout status of healthcare workers. These countries were geographically distributed in the United States, Spain, China, Italy, Taiwan, France, Canada, Malaysia, South Korea, and the United Kingdom, showing the scientific production by country (Figure 3). This map was created *via* “Biblioshiny,” a web interface of the bibliometric package. The various blue shades represent diverse levels of productivity. Consequently, if the blue shades darker means greater the productivity. The three most productive countries were the United States (US) (*n* = 26), Spain (*n* = 20), and China (*n* = 17).

### Top 5 most global and cited publications (2020-2021-2022)

The study investigated the global and local citations from publications. The number of article citations obtained across the entire database is the international source. In contrast, the local cisourceseasure is the number of citasourcesaper obtained and the data included in the survey (Aria and Cuccurullo, 2017). Tables 1–3 show the fifteen most cited publications globally and locally from 2020 until 2022. It is essential to mention that the rankings are based on local citations.

**Table 1:** Top 5 most global and local cited publications (2020).

**References**	**Title**	**Source title**	**Year**	**Cited**
Wu et al. (2020)	A comparison of burnout frequency among oncology physicians and nurses working on the frontline and usual wards during the COVID-19 epidemic in Wuhan, China	Journal of Pain and Symptom Management	2020	218
Buselli et al. (2020)	Professional quality of life and mental health outcomes among health care workers exposed to SARS-CoV-2 (COVID-19)	International Journal of Environmental Research and Public Health	2020	100
Marvaldi et al. (2020)	Anxiety, depression, trauma-related, and sleep disorders among healthcare workers during the COVID-19 pandemic: A systematic review and meta-analysis	Neuroscience and Biobehavioral Reviews	2020	55
Duarte et al. (2020)	Burnout among Portuguese healthcare workers during the COVID-19 pandemic	BMC Public Health	2020	43
Ruiz-Fernández et al. (2020)	Quality of life in nursing professionals: Burnout, fatigue, and compassion satisfaction	International Journal of Environmental Research and Public Health	2020	41

**Table 2:** Top 4 most global and local cited publications (2021).

**References**	**Title**	**Source title**	**Year**	**Cited**
Chen et al. (2021)	Risk factors for depression and anxiety in healthcare workers deployed during the COVID-19 outbreak in China	Social Psychiatry and Psychiatric Epidemiology	2021	38
Yörük and Güler (2021)	The relationship between psychological resilience, burnout, stress, and sociodemographic factors with depression in nurses and midwives during the COVID-19 pandemic: A cross-sectional study in Turkey	Perspectives Psychiatric Care	2021	34
Jalili et al. (2021)	Burnout among healthcare professionals during COVID-19 pandemic: a cross-sectional study	International Archives of Occupational and Environmental Health	2021	27
Sirois and Owens (2021)	Factors associated with psychological distress in health-care workers during an infectious disease outbreak: a rapid systematic review of the evidence	Frontiers in Psychiatry	2021	24

**Table 3:** Top 5 most global and local cited publications (2022).

**References**	**Title**	**Source title**	**Year**	**Cited**
Lange et al. (2022)	Impact on the mental health of the COVID-19 outbreak among general practitioners during the sanitary lockdown period	Irish Journal of Medical Science	2022	3
Acar Sevinc et al. (2022)	Anxiety and burnout in anesthetists and intensive care unit nurses during the COVID-19 pandemic: a cross-sectional study	Brazilian Journal of Anesthesiology (English Edition)	2022	2
Sikaras et al. (2021)	Nursing staff fatigue and burnout during the COVID-19 pandemic in Greece	AIMS Public Health	2022	2
Balakrishnan et al. (2022)	COVID-19 depression and its risk factors in the Asia Pacific – A systematic review and meta-analysis	Journal of Affective Disorders	2022	1
Bertuzzi et al. (2022)	Longitudinal survey on the Psychological Impact of the COVID-19 Pandemic on Healthcare Workers (PsyCOVer) in France: Study protocol	BMJ Open	2022	1

The three dominant global articles cited were “A Comparison of Burnout Frequency Among Oncology Physicians and Nurses Working on the Frontline and Usual Wards During the COVID-19 Epidemic in Wuhan, China” (n: 218) in 2022 (Wu et al., 2020). It is followed by an article in 2021 entitled “Anxiety, Depression, Trauma-Related, and Sleep Disorders Among Healthcare Workers During the COVID-19 Pandemic: A Systematic Review and Meta-Analysis” (n: 55) (Marvaldi et al., 2021). Then, in 2022, the third one is entitled “Impact on the Mental Health of the COVID-19 Outbreak Among General Practitioners During the Sanitary Lockdown Period” (n: 3) (Lange et al., 2022). The following are the Top 5 most global and locally referenced publications (Table 1).

Among the fifteen most cited papers analyzed was identifying the studies comparing burnout frequency between doctors and nurses at the forefront by looking at the relationship between the factors and different variables that related to the nurse retention and physician. The healthcare group had a lower frequency of fatigue (13 vs. 39%; *P* < 0.0001) and was less worried about being infected than the healthcare staff (Wu et al., 2020). Another study discovered that health personnel has been dealing with the COVID-19 epidemic, which has resulted in many critical patients, deaths, and a heavy burden. Furthermore, the mental health of healthcare workers has an impact on service quality. The frequency of mental health disorders among healthcare workers during the pandemic was evaluated by PRISMA's systematic review and meta-analysis. The following combined prevalence was estimated: 300% anxiety (95% CI, 24.2–37.05); 311% depression (95% CI, 25.7–36.8); 565% acute stress (95% CI - 30.6–80.5); 20.2% post-traumatic stress (95% CI, 9.9–33.0); 44.0% sleep disturbance (95% CI, 24.6–64.5) (Marvaldi et al., 2021). In addition, another study aimed to verify the prevalence of burnout in health workers to identify its specific determinants.

The background of the COVID-19 outbreak may influence mental health, particularly among healthcare personnel. The study's goal was to see how COVID-19 affected the mental health of French general practitioners (GPs). During the initial shutdown, a postal-based survey was undertaken. Stress, post-traumatic stress symptoms, exhaustion, and self-efficacy were assessed using four psychologically validated self-report questionnaires: Perceived Stress scale, revised Impact Scale, Maslach Fatigue Inventory, and General Self-Efficacy scale. The sample consisted of 332 general practitioners (43.50% women, mean age = 50.74 ± 11.91). General practitioners working in high epidemic areas represented 27.71% of the sample (*n* = 92).

Next (Lange et al., 2022), symptoms of high burnout were identified and found in 79 (24.46%), 137 (42.41%), and 17 (5.26%) participants. Only general self-efficacy scores differed significantly according to the status of the epidemic location, with lower scores in general practitioners working in high epidemic locations (33.37 ± 4.64 vs. 32.06 ± 5.43; *P* = 0.04). In addition, women were revealed to have more stress and fatigue symptoms than men (*P* = 0.01). COVID-19 had a psychological impact on healthcare professionals during the sanitation shutdown, including exhaustion and post-traumatic stress symptoms, according to the studies.

### Mapping of the ten most frequent authors' keywords and keywords plus

**Figure 4:**
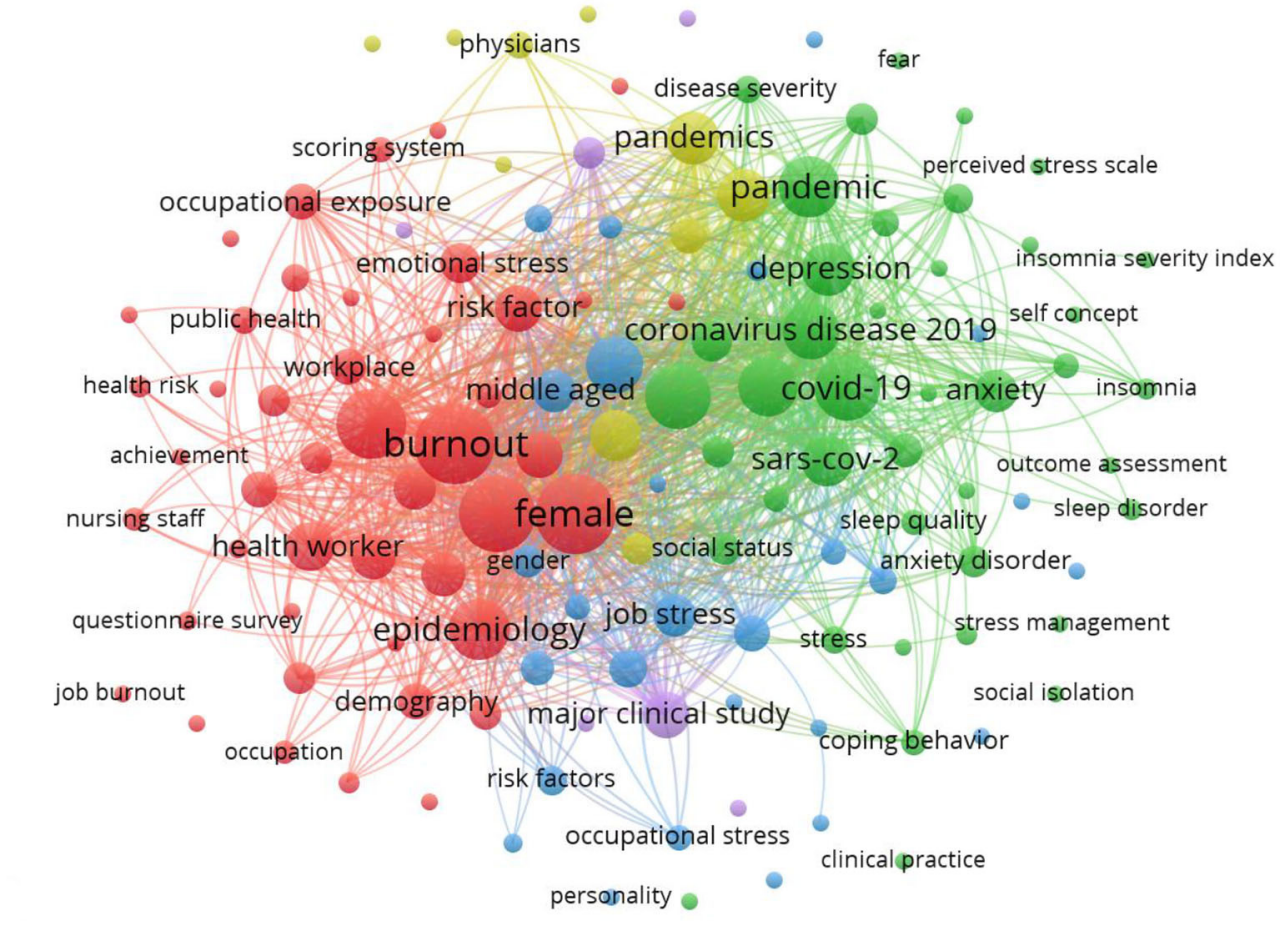
Mapping authors' keywords.

Table 4 and Figure 4 display the most frequent keyword mappings from the author and the database (plus keywords). The most common keywords used by the authors were “Health Worker (*n* = 2,991), “burnout” (*n* = 2,750), followed by “Epidemiology” (*n* = 1,748). Contrastingly, the keyword provided by the database with a bigger frequency was “Human” (*n* = 3,127), followed by “female” (*n* = 2,991). The keywords chosen should reflect the entire research topic and can be used to identify a trend for future research (de Oliveira et al., 2021; Lange et al., 2022). Therefore, as long as they bring an overview of the aspects covered in the study, the keywords, among others, comprised stress, love saying fatigue, job satisfaction, and work fatigue.

**Table 4:** Mapping the author's 10 keywords and most often keywords.

**Ranking**	**Authors' keywords**	**Frequency**	**Ranking**	**Author's plus**	**Frequency**
1**°**	Health worker	2,991	11**°**	Humans	3,127
2**°**	Burnout	2,750	12**°**	Female	2,991
3**°**	Epidemiology	1,748	13**°**	Male	2,770
4**°**	Depression	1,344	14**°**	Adult	2,729
5**°**	Job burnout	1,303	15**°**	Personality	566
6**°**	Workplace	1,269	16**°**	Stress management	371
7**°**	Risk factor	1,083	17**°**	Occupational stress	317
8**°**	Job stress	922	18**°**	Social status	312
9**°**	Stress	381	19**°**	Nursing staff	294
10**°**	Emotional stress	831	20**°**	Middle aged	176

### Trending topics

Table 5 presents the trending topics. The size or diameter represents the frequency of phrases provided by the author in keywords, based on bibliography periodicity parameters of at least five words each year. In 2020–2022, terms such as burnout (*n* = 3,269), health (*n* = 2,399), Healthcare (*n* = 1,435), workers (*n* = 1,396), and stress (*n* = 1,318) can be observed in Table 5. Given that many healthcare personnel work closely with individuals affected by the disease, the study results may reflect the influence of this situation. Furthermore, recent studies have shown that, in the face of a pandemic, some health professionals experience fatigue (World Health Organization, 2020) and psychological distress (Zhang et al., 2020; de Oliveira et al., 2021). The research gap is a lack of understanding of how the pandemic might affect the mental condition of health practitioners (Duarte et al., 2020).

**Table 5:** Trending topic of healthcare workers' burnout.

**Word**	**Length**	**Count**	**Weighted percentage (%)**	**Word**	**Length**	**Count**	**Weighted rate (%)**
Burnout	7	3,269	1.35	Factors	7	501	0.21
Health	6	2,399	0.99	Support	7	488	0.20
COVID	5	2,390	0.99	Public	6	487	0.20
Healthcare	10	1,435	0.59	Associated	10	486	0.20
Workers	7	1,396	0.58	Resilience	10	462	0.19
Stress	6	1,318	0.54	Impact	6	427	0.18
Pandemic	8	1,175	0.48	Emotional	9	406	0.17
Anxiety	7	1,018	0.42	Coronavirus	11	379	0.16
Psychological	13	986	0.41	Exhaustion	10	360	0.15
Care	4	835	0.34	Prevalence	10	360	0.15
Depression	10	796	0.33	Physicians	10	359	0.15
Work	4	718	0.30	Staff	5	351	0.14
Symptoms	8	712	0.29	Distress	8	345	0.14
Patients	8	699	0.29	Disease	7	340	0.14
Mental	6	677	0.28	Years	5	337	0.14
Hospital	8	642	0.26	Professionals	13	321	0.13
Medical	7	595	0.25	Results	7	318	0.13
Job	3	570	0.23	Sars	4	315	0.13
Data	4	563	0.23	Found	5	301	0.12
Nurses	6	551	0.23	Well	4	300	0.12
Levels	6	543	0.22	Sample	6	294	0.12
Working	7	535	0.22	Social	6	293	0.12
Personal	8	532	0.22	Emergency	9	289	0.12
Risk	4	521	0.21	General	7	285	0.12
Participants	12	503	0.21	Professional	12	283	0.12

Table 6 reveals the Pearson correlation between the total burnout scale scores on health workers, professionals, exhaustion, mental, stress, personal, depression, symptoms, emergency, system, job, and impact. The top results showed that overall burnout scores were positively associated with anxiety, depression, and stress symptoms, with correlation values ranging from 0.84 to 0.73, according to the point-biserial correlation coefficient. In addition, job burnout had a significant relationship with exhaustion at 0.89; depression r = 0.73, impacting a score of 0.66. At the same time, professional fulfillment and interpersonal disengagement showed a finite correlation (r-value between 0.86 and 0.77). There were relationships between burnout scores, sociodemographic traits, work-related factors, and psychological wellbeing.

**Table 6:** Relation of burnout status of healthcare workers study topics.

	**Code A**	**Code B**	**Pearson correlation coefficient**
Burnout of Healthcare Workers	Burnout	Professional	0.819856
	Burnout	Exhaustion	0.806159
	Burnout	Mental	0.803155
	Burnout	Stress	0.800804
	Burnout	Personal	0.746487
	Burnout	Depression	0.728673
	Burnout	Symptoms	0.72084
	Burnout	Emergency	0.706716
	Burnout	System	0.705677
	Burnout	Job	0.702803
	Burnout	Impact	0.677516

Overall, healthcare employees around the world reported having a distress high level of psychological distress. Consistent with previous research, these recent findings imply that COVID-19 is a unique circumstance influencing the psychological health of healthcare workers in general. It is known that since the pandemic started, the medical personnel has struggled to fight the epidemic on the front lines and protect public health. Healthcare workers suffer a significant increase in workload and the backbone of the struggle in the first line of epidemic prevention and control is a high risk of infection and occupational stress (Conti et al., 2021).

This study indicates that the correlation coefficient is point-biserial, meaning that with correlation values ranging from 0.84 to 0.73, burnout scores are positively associated with anxiety, sadness, and stress symptoms. Job burnout has a significant relationship with exhaustion 0.89; depression r = 0.73, impacting a score of 0.66. At the same time, professional fulfillment and interpersonal disengagement showed a finite correlation (r-value between 0.86 and 0.77). The table shows the correlation between burnout scores, sociodemographic characteristics, work-related factors, and psychological wellbeing.

In this recent study, when compared to persons who did not experience burnout, burnout was connected with higher workload, psychological distress symptoms, and a reduction in personal resources. This hypothesis has been entirely confirmed. In line with previous research (Chou et al., 2014; Barello et al., 2020), the researchers found that the healthcare workers directly in charge of dealing with COVID-19 pandemic patients were more likely to experience depression, stress, and burnout compared to healthcare staff who were not personally involved in the treatment medical work. This could be because lower-level jobs have less control over procedures and decision-making capabilities than higher-level jobs. In addition, several authors have pointed out the main differences between professions regarding the symptoms evaluated during the COVID-19 pandemic applied to healthcare workers and other functions, such as doctors and nurses presenting more symptoms of anxiety and depression (Pappa et al., 2020). This difference is related to the contact of these professionals with infected patients.

**Figure 5:**
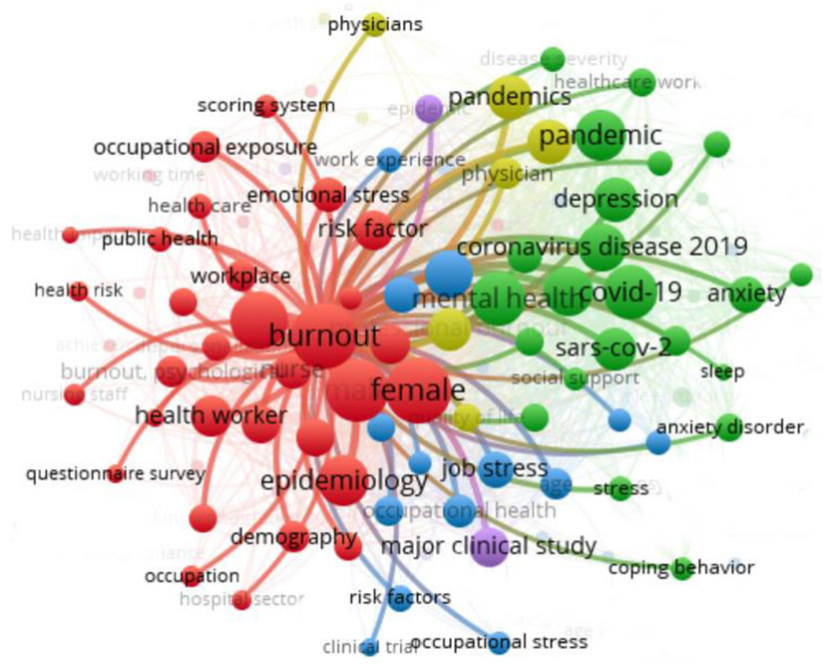
Visualization of the correlation between burnout and health workers.

In addition, our findings found those with high levels of burnout worked more on the front lines, lost more patients, had fewer personal resources, and reported more sadness, anxiety, and post-traumatic symptoms than those with low levels of burnout. Thus, tiredness is associated with the professional role (being part of a healthcare workforce and clinical practice working on the front line). This study finding proves the previous studies that burnout is an essential driver of healthcare workers burnout. It can be seen from the visualization of the correlation between burnout with health workers (Figure 5). Moreover, this research will be a follow-up study to assess work ability and job satisfaction using Work Ability Index (WAI).

The visualization depicts many responsibilities of health workers explained in this relationship, including long-term workload, lack of sleep, depression, and stress. Healthcare workers with burnout showed more workload features, symptoms of psychological distress, and lower personal resources than those who did not experience burnout. This hypothesis is fully confirmed. Kami found that health workers on direct duty to treat COVID-19 pandemic patients were affected by depression, stress, and burnout compared to health staff who were not personally involved in medical work. This result may be because, in lower-level work, control over procedures and decision-making capacity is lower than in other higher-level positions. Some authors have pointed out that the main differences between professions regarding the symptoms evaluated during the COVID-19 pandemic apply to health workers and different situations, such as doctors and nurses presenting more symptoms of anxiety and depression. This difference is related to the contact of these professionals with infected patients. Although workloads are different, the medical staff must think about workload. This study will be a follow-up study by looking at the level of fatigue of medical staff during the COVID-19 pandemic.

## Conclusion

Based on the presentation of the findings above, the research can be concluded. *First*, there are many studies on burnout of health workers during the pandemic; 150 documents were published during the 2020–2022 timeframe. It confirms that the pandemic has not only diverted the economy and socio-politics, but the most critical thing to distract the world is the health side. Furthermore, 2021 is when studies on burnout health workers during the pandemic had the most trends. In 2021, around 80,000 to 180,000 health workers worldwide died from COVID-19. Most of them were doctors and nurses. Most health workers also died due to various sources of psychological stress, such as uncertainty about disease progression (short-term and long-term effects), medication, lack of personal protective equipment (PPE), physical exhaustion, excessive workload, and worry about direct exposure to COVID-19 in the workplace. This paper provides lessons that the presence of a pandemic informs future government and health council decisions about allocating resources for the welfare of health workers and better reports how these resources can be targeted to those at highest risk for adverse mental health outcomes. Furthermore, burnout during the pandemic is associated with decreased quality of care and patient safety. Efficient medical personnel fatigue management has practical implications for employees in the medical sector and patients, with significant consequences for how the health system responds to the current outbreak. In the future, health workers need to think carefully about their workload or resources.

*Second*, the findings in this study revealed that the Pearson correlation between the total burnout scale scores on health workers, professionals, exhaustion, mental, stress, personal, depression, symptoms, emergency, system, and job impact indicated that the overall burnout score was related to anxiety and depression. From the findings of this study, it was found that one of the causes of the large number of health workers dying was influenced by the level of fatigue. They must be taking action on the workload standards for the healthcare workers. In addition, stress symptoms had correlation values ranging from 0.84 to 0.73. Job burnout also had a significant relationship with exhaustion at 0.89; depression r = 0.73, impacting a score of 0.66. At the same time, the fulfillment of professional and interpersonal disengagement showed a Pearson correlation between the total burnout scale scores on health workers, professionals, exhaustion, mental, stress, personal, depression, symptoms, emergency, system, job, and impact. Overall, participants in health care workers worldwide experienced high levels of psychological distress. We also found that healthcare workers directly in charge of dealing with COVID-19 pandemic patients were more likely to experience depression, stress, and burnout was higher among healthcare workers who were not directly involved in medical care.

## Data availability statement

The original contributions presented in the study are included in the article/supplementary material, further inquiries can be directed to the corresponding author.

## Author contributions

MU: conceptualization, data collection, analysis, and editing final draft. MA: writeup, review, and editing. SA: data analysis, methodology, and review. All authors contributed to the article and approved the submitted version.

## Conflict of interest

The authors declare that the research was conducted in the absence of any commercial or financial relationships that could be construed as a potential conflict of interest.

## Publisher's note

All claims expressed in this article are solely those of the authors and do not necessarily represent those of their affiliated organizations, or those of the publisher, the editors and the reviewers. Any product that may be evaluated in this article, or claim that may be made by its manufacturer, is not guaranteed or endorsed by the publisher.
